# Cancer Cells Promote Phenotypic Alterations in Hepatocytes at the Edge of Cancer Cell Nests to Facilitate Vessel Co-Option Establishment in Colorectal Cancer Liver Metastases

**DOI:** 10.3390/cancers14051318

**Published:** 2022-03-04

**Authors:** Miran Rada, Migmar Tsamchoe, Audrey Kapelanski-Lamoureux, Nour Hassan, Jessica Bloom, Stephanie Petrillo, Diane H. Kim, Anthoula Lazaris, Peter Metrakos

**Affiliations:** Cancer Research Program, Research Institute of the McGill University Health Centre, Montreal, QC H4A 3J1, Canada; migmar.tsamchoe@mail.mcgill.ca (M.T.); audrey.kapelanski-lamoureux@mail.mcgill.ca (A.K.-L.); nour.hassan@mail.mcgill.ca (N.H.); jessica.bloom@mail.mcgill.ca (J.B.); stephanie.petrillo@muhc.mcgill.ca (S.P.); hyunbin.kim@mail.mcgill.ca (D.H.K.); anthoula.lazaris@mail.mcgill.ca (A.L.)

**Keywords:** vessel co-option, hepatocytes, apoptosis, autophagy, EMT, motility

## Abstract

**Simple Summary:**

Tumour cells in colorectal cancer liver metastases (CRCLM) obtain their blood supply via two major mechanisms: (i) sprouting angiogenesis, through the generation of new vessels; (ii) vessel co-option, where the cancer cells hijack the pre-existing vasculature. The current treatment for CRCLM targets angiogenesis; however, these treatments are ineffective on cancer cells utilizing vessel co-option to gain their blood supply. Our study suggests that cancer cells stimulate phenotypic alterations in the cells of surrounding liver tissue (hepatocytes) in vessel co-opting lesions. These modifications facilitate cancer cells to infiltrate through the liver tissue and hijack the pre-existing vasculature to obtain oxygen and nutrients.

**Abstract:**

Vessel co-option is correlated with resistance against anti-angiogenic therapy in colorectal cancer liver metastases (CRCLM). Vessel co-opting lesions are characterized by highly motile cancer cells that move toward and along the pre-existing vessels in the surrounding nonmalignant tissue and co-opt them to gain access to nutrients. To access the sinusoidal vessels, the cancer cells in vessel co-opting lesions must displace the hepatocytes and occupy their space. However, the mechanisms underlying this displacement are unknown. Herein, we examined the involvement of apoptosis, autophagy, motility, and epithelial–mesenchymal transition (EMT) pathways in hepatocyte displacement by cancer cells. We demonstrate that cancer cells induce the expression of the proteins that are associated with the upregulation of apoptosis, motility, and EMT in adjacent hepatocytes in vitro and in vivo. Accordingly, we observe the upregulation of cleaved caspase-3, cleaved poly (ADP-ribose) polymerase-1 (PARP-1) and actin-related protein 2/3 (ARP2/3) in adjacent hepatocytes to cancer cell nests, while we notice a downregulation of E-cadherin. Importantly, the knockdown of runt-related transcription factor 1 (RUNX1) in cancer cells attenuates the function of cancer cells in hepatocytes alterations in vitro and in vivo. Altogether, our data suggest that cancer cells exploit various mechanisms to displace hepatocytes and access the sinusoidal vessels to establish vessel co-option.

## 1. Introduction

Colorectal cancer (CRC) is the third most common diagnosed cancer and the second leading cause of cancer death worldwide [1]. Metastases account for most of the CRC-related death [2,3]. At the time of diagnosis, approximately 25% of patients with CRC are diagnosed with synchronous metastases [4]. Additionally, 30–40% of the patients are found to develop metachronous metastases and another 25–30% of the patients develop liver metastases during the follow-up [4]. Currently, surgical resection is the main treatment approach for CRCLM patients, which can achieve 5-year survival rates above 50%. However, only 20% of the patients are qualified for upfront surgery and the rest are left with palliative options [5,6]. 

Two major histopathological heterogeneity have been described in CRCLM, including the desmoplastic histopathological growth pattern (DHGP) and replacement histopathological growth pattern (RHGP) [7,8,9,10]. In DHGP lesions, the cancer cells are prevented from direct contact with hepatocytes by a desmoplastic rim and obtain their blood supply through angiogenesis [8,10]. In contrast, the cancer cells in RHGP lesions are in direct contact with hepatocytes due to the lack of a desmoplastic ring and obtain their blood supply through the co-option of the existing vasculature, i.e., the tumour incorporates pre-existing blood vessels instead of promoting angiogenesis [8,10,11,12].

The liver vascular architecture is characterized by sinusoidal vessels. In order to access the sinusoidal vessels, the cancer cells must invade the liver parenchyma [8,11], as well as displace the hepatocytes around sinusoidal vessels and occupy their space [13]. We previously hypothesized that the cancer cells could form a unique microenvironment to induce hepatocyte displacement through different pathways, such as apoptosis, necrosis, and/or autophagy, as well as an elevated motility and epithelial–mesenchymal transition (EMT) [13]. However, the molecular mechanisms involved in hepatocytes displacement are not yet clearly defined. 

Apoptosis is a type of programmed cell death characterized by various energy-dependent biochemical events resulting in changes in cellular morphology and death [14]. The caspase (e.g., caspase-3)-mediated cleavage of poly (ADP-ribose) polymerase-1 (PARP-1) cleavage is recognized as a hallmark of apoptosis [15,16]. Apoptosis plays an essential role in tumour development and cancer metastases [17,18,19,20]. The evasion of cancer cells from apoptosis is associated with resistance to therapeutic agents [18,21]. It is still unclear whether apoptosis is involved in the development of vessel co-option, specifically in CRCLM. However, a differential expression of apoptotic genes has been reported between angiogenic and nonangiogenic lesions in non-small-cell lung cancer (NSCLC) [22,23]. Accordingly, the angiogenic lesions expressed higher levels of antiapoptotic genes, including ANXA7 and SOD1, while the proapoptotic genes, including FOS, FAH, and PRODH, were upregulated in nonangiogenic lesions [22]. 

Another candidate mechanism of hepatocyte displacement by cancer cells in vessel co-opting CRCLM is autophagy [13]. Autophagy, a “self-eating” phenomenon, is an intracellular degradative pathway to maintain cell function and survival [24]. Autophagy plays a critical role in liver protection against injury and steatosis [25]. Autophagy is known to induce tumour development and drug resistance in the liver [24]. Despite the ample evidence for a positive correlation between autophagy with antiangiogenic (e.g., bevacizumab) therapy resistance in various cancers [26,27], the role of autophagy in the establishment of vessel co-opting tumours is still unclear. 

EMT is another potential mechanism that may expedite hepatocyte displacement in vessel co-opting CRCLM [13]. EMT comprises multiple biochemical modifications to allow epithelial cells to develop a mesenchymal cell phenotype, such as an elevated migration capacity and the production of extracellular matrix (ECM) components [28]. It has been reported that EMT in cancer cells contributes to tumour progression, metastases, and resistance to cancer treatment [29]. Indeed, EMT plays an important role in promoting cancer cell motility [30,31]. We recently demonstrated the upregulation of EMT and motility biomarkers in the cancer cells of vessel co-opting CRCLM lesions, which correlated with the upregulation of transforming growth factor beta 1 (TGFβ1) in their proximate liver parenchyma [32]. It is worth mentioning that the role of EMT in the cells of the organ that hosts tumours is poorly investigated. In injured livers, hepatocytes have been shown to undergo EMT [33], which is promoted mainly through TGFβ1 [34].

In the current study, we explored the contribution of apoptosis, autophagy, EMT, and motility in hepatocyte displacement by cancer cells in vessel co-opting CRCLM. Our results propose that the cancer cells in the tumour–liver interface of co-opting lesions stimulate the displacement of the hepatocytes through the upregulation of proteins that are associated with high levels of apoptosis, motility, and EMT in hepatocytes and, consequently, occupy their space to co-opt the sinusoidal blood vessels. Thus, our results indicate that the metastatic cancer cells alter the hepatocytes phenotype in vessel co-opting CRCLM lesions to support their co-option of the pre-existing blood vessels. 

## 2. Methods

### 2.1. Patient Samples

This study was approved by the McGill University Health Centre Institutional Review Board. All patients signed the informed consent. 

### 2.2. Cells Culturing and Treatment

The cells were cultured at 37 °C with 5% CO_2_ in DMEM (Wisent Inc., St-Bruno, QC, Canada, #319-005-CL) supplemented with 10% FBS (Wisent Inc., #085-150) and 1% of streptomycin penicillin/streptomycin (10,000 units/mL of penicillin and 10,000 µg/mL) (Wisent Inc., 450-201-EL). 

Indirect coculturing was performed with 6-well inserts (Falcon, Glendale, AZ, USA, #353090) and companion plates (Falcon, Glendale, AZ, USA, #353502) as described previously [32]. The cells were seeded in DMEM supplemented with 10% FBS and 1× penicillin/streptomycin overnight. The next day, the growth medium was replaced with serum-free DMEM, and the cells were incubated for 48 h at 37 °C. 

The treatment of IHH cells with recombinant TGFβ1 was performed as published previously [32]. Briefly, the IHH cells were grown as mentioned above. After 24 h, the conditioned media was replaced with serum-free DMEM media supplemented with recombinant TGFβ1 (Peprotech, Cranbury, NJ, USA, # 100-21) and incubated for 24 h at 37 °C. 

### 2.3. Lentiviral shRNA Knockdown

The lentiviral vectors were produced in 293T cells using the calcium phosphate method [32,35]. We used the following constructs: Scrambled shRNA#: SHC016, RUNX1#1: TRCN0000338428. A monolayer of HT29 cancer cells was prepared as mentioned above. After 24 h, the lentivirus-containing medium supplemented with 8 µg/mL of polybrene was added to HT29 cancer cells and incubated for 72 h at 37 °C with 5% CO_2_, followed by treatment with 1 µg/mL of Puromycin (Wisent Inc., St-Bruno, QC, Canada, 450-162-XL). 

### 2.4. Immunoblotting

After harvesting the cells, the lysates were prepared by resuspending the cells in RIPA buffer (Sigma Aldrich, Oakville, ON, Canada, #R0278) supplemented with protease inhibitor (Sigma Aldrich, #4693124001). To rupture the cells, the resuspended cells passed through a 25 G needle 15 times. The protein content in the samples was measured using BCA Protein Assay Kit (Thermo Scientific, Saint-Laurent, QC, Canada, #23225). The generated lysates were used for immunoblotting with following antibodies: GAPDH 1:2000 (Abcam, Waltham, MA, USA, #ab9485), cleaved caspase-3 1:500 (Cell signaling, # 9664S), cleaved PARP-1 1:500 (Cell signaling, # 5625S), ARP2/3 1:1000 (Millipore, #MABT95), Vimentin 1:1000 (Abcam, Waltham, MA, USA, #ab16700), RUNX1 1:500 (LS Bio, Seattle WA, USA, #LS-C353932), TGFβ1 1:500 (Abcam, Waltham, MA, USA, #ab215715). 

### 2.5. Immunohistochemical Staining

Ten independent formalin-fixed paraffin-embedded (FFPE) CRCLM sections (*n* = 5 RHGP; *n* = 5 DHGP) were procured and used for each staining as a biological replicate. In brief, the sections were deparaffinized with xylene (Leica, Heerbrugg, Switzerland, #3803665) followed by hydration with ethanol (Greenfield, Boucherville, QC, Canada, #P016EAAN) and then with distilled water. Then, the sections were exposed to antigen retrieval, as well as endogenous peroxidase inhibition (Dako, #S2003). The sections were incubated with 1% goat serum buffer for 1 h followed by primary antibody in 1% goat serum overnight at 4 °C. The next day, the sections were washed and incubated with secondary antibodies (Dako, Burlington, ON, Canada, anti-mouse: #K4001; anti-rabbit: #K4003) for 1 h. The positive signals were stained with diaminobenzidine (DAB) substrate (Dako, Burlington, ON, Canada, #K3468). The sections were scanned at 20× magnification (Aperio ScanScope XT System) and the images were analyzed with Aperio ImageScope ver.11.2.0.780 as published previously [10,11,12]. 

The following primary antibodies were used: cleaved caspase-3 1:100 (Cell signaling, Whitby, ON, Canada, # 9664S), cleaved PARP-1 1:50 (Cell signaling, Whitby, ON, Canada, # 5625S), E-Cadherin 1:200 (Cell signaling, Whitby, ON, Canada, # 3195S; R&D systems, Toronto, ON, Canada, #MAB1838-100), ARP2/3 1:300 (Millipore, Etobicoke, ON, Canada, #MABT95; Bioss, Laval, QC, Canada, #bs-12524R), RUNX1 1:200 (LS Bio, Seattle WA, USA, #LS-C353932).

### 2.6. Immunofluorescence Staining

We performed immunofluorescent (IF) staining for specimens as described previously [32]. In brief, the sections were deparaffinized with xylene (Leica, #3803665), hydrated with ethanol (Comalc, #P016EAAN), as well as distilled water. Antigen retrieval was performed followed by blocking with 1% goat serum buffer (1 h incubation at room temperature) and primary antibody (overnight incubation at 4 °C). The sections were washed thrice and incubated with secondary antibody 1:1000 (Alexa Flour 594 goat anti-rabbit IgG and Alexa Flour 488 goat anti-mouse IgG (Invitrogen, Burlington, ON, Canada, #A11037 and #A10680, respectively)) for 2 h at room temperature, followed by washing. The sections were incubated with DAPI 1:1000 (Thermo Fisher Scientific, Saint-Laurent, QC, Canada, D1306) for 10 min and covered with cover glass using ProLong Gold Antifade Mountant (Thermo Fisher Scientific, Saint-Laurent, QC, Canada, P36934). Six independent tumour specimens, including RHGP (*n* = 3) and DHGP (*n* = 3), were procured and used for each staining as a biological replicate.

We performed IF staining for cultured cells, which followed the protocol [32]. In brief, the cells were fixed with 4% paraformaldehyde (Biolegend, San Diego, CA, USA, #420801), permeabilized with 0.1% Triton X-100 (Bio-Rad, Saint-Laurent, QC, Canada, #161-0407), blocked with 5% BSA (GE Healthcare Life Science, Chicago, IL, USA, #SH30574.02), and incubated with primary antibody prepared with 5% BSA at 4  °C overnight. The next day, the cells were washed and incubated with secondary antibodies 1:1000 (Alexa Flour 594 goat anti-rabbit IgG and Alexa Flour 488 goat anti-mouse IgG (Invitrogen, Burlington, ON, Canada, #A11037 and #A10680, respectively)) for 2 h. The cells were then incubated with DAPI 1:1000 for 10 min and covered with coverslips using ProLong Gold Antifade Mountant (Thermo Fisher Scientific, Saint-Laurent, QC, Canada, #P36934). 

The sections were examined with Zeiss LSM780 confocal microscope. The intensity of positive signals was analyzed using ImageJ (NIH, Bethesda, MD) software. Average pixel intensity was measured from three randomly selected areas for each sample. We used these antibodies: HSA 1:300 (Santa Cruz, Dallas, TX, USA, #SC5893), cleaved caspase-3 1:100 (Cell signaling, # 9664S), cleaved PARP-1 1:50 (Cell signaling, # 5625S), ARP2/3 1:300 (Millipore, #MABT95), Vimentin 1:200 (Abcam, ab16700), CK20 (Thermo Fisher, Saint-Laurent, QC, Canada, #MA5-13263), E-Cadherin 1:200 (Cell signaling, # 3195S). 

### 2.7. Scratch Assay

The cells were seeded in a treated plate with poly-L-Lysine (Millipore, #A-005-CL) and incubated with DMEM supplemented with 10% FBS and 1% penicillin/streptomycin at 37  °C overnight. The next day, the medium was removed and a wound was introduced into the monolayer cells using a p200 pipette tip (Kinesis, Montreal, QC, Canada, #TF-200-R-S) [11,32]. The cells were washed with PBS and the stripped areas were photographed (0 h) and incubated for 24 h at 37 °C with serum-free DMEM. The cells were washed with PBS and the stripped areas were snapped (24 h). The wound opening was analyzed with ImageJ (NIH, Bethesda, MD). 

### 2.8. MTT Assay

IHH hepatocytes and cancer (LS174, SW620 or HT29) cells were seeded at a density of 500,000 cells/well in 6-well companion plates and inserts, respectively. The cells were cultured in DMEM supplemented with 10% FBS and 1% penicillin/streptomycin at 37 °C with 5% CO_2_. The next day, the conditioned media were replaced with serum-free DMEM media and the cells were incubated at 37 °C. After 48 h, the media was aspirated and MTT assay (Abcam, #Ab211091) was performed according to the manufacturer’s protocol. Briefly, cells were incubated with 500 μL serum-free DMEM media and 500 μL MTT reagent and incubated the plate at 37 °C for 3 h. After incubation, we added 1500 µL of MTT solvent into each well and incubated for 15 min. Absorbance was, subsequently, measured at 590 nm using the Tecan Ultra plate reader (Tecan, Germany).

### 2.9. Statistical Analysis

Data were analyzed with GraphPad Prism software version 7.0 (GraphPad Software, CA, USA). Data presented as mean ± standard deviation. Unpaired Student’s *t*-test was used to determine a significant difference between the means of the two groups. A *p*-value of less than 0.05 was considered significant.

## 3. Results

### 3.1. Upregulation Pression of Proapoptotic Biomarker Vessel Co-Opting Lesions

**Figure 1:**
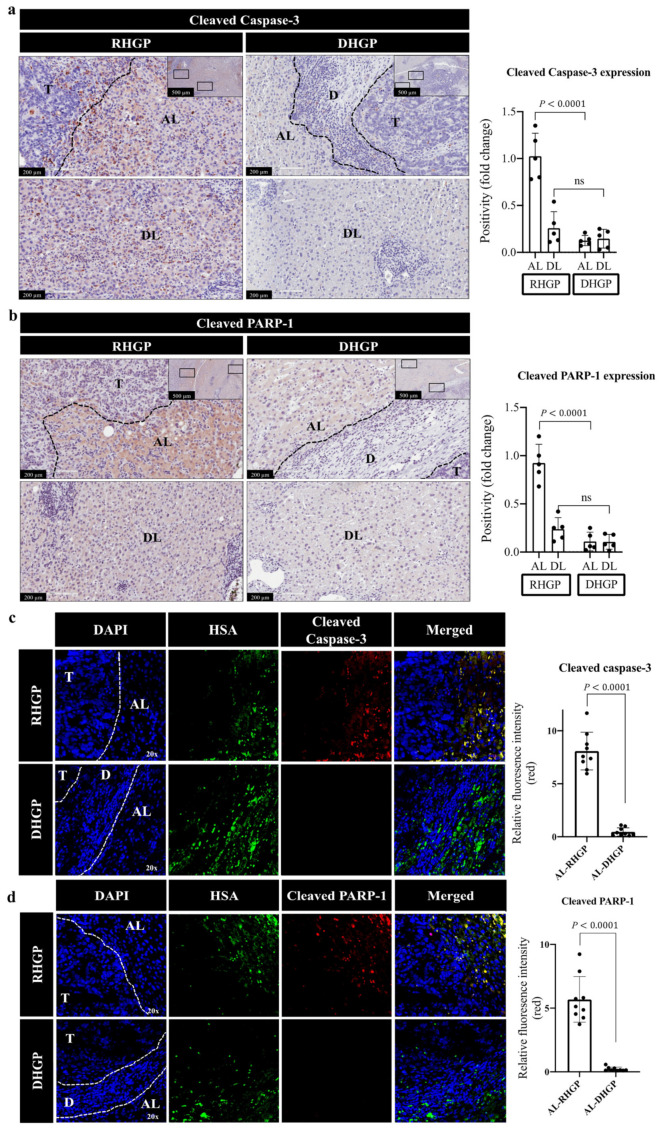
Overexpression of proapoptotic biomarkers in vessel co-opting CRCLM. (**a**,**b**) Immunohistochemical staining of chemonaive CRCLM lesions using cleaved caspase-3 and cleaved PARP-1 antibodies, respectively (left panel). The right panels represent quantification of staining positivity that assessed in RHGP (*n* = 5) and DHGP (*n* = 5) lesions using Aperio software. (**c**,**d**) co-IF of chemonaive CRCLM lesions showing HSA (green) and cleaved caspase-3 (red) or cleaved PARP-1 (red). The right panels represent the quantification of positive pixels in CRCLM specimens, including RHGP (*n* = 3) and DHGP (*n* = 3). Average pixel intensity was measured from three randomly selected areas for each sample. Data are presented as the mean ± SD. ns = Not significant. AL—adjacent liver; D—desmoplastic ring; DL—distal liver; T—tumour.

Hepatocyte replacement by cancer cells was frequently observed at the edge of cancer cell nests in vessel co-opting RHGP lesions, which allowed the cancer cells to take over the hepatocyte-occupied space to hijack the sinusoids and obtain a blood supply [8,10,13]. However, the mechanistic pathways underlying hepatocyte replacement by cancer cells are poorly understood. We postulated that apoptosis may contribute to hepatocyte replacement by cancer cells. To test this possibility, we performed immunohistochemical staining for chemonaive CRCLM specimens (*n* = 5 DHGP; *n* = 5 RHGP) using proapoptotic biomarkers, including cleaved caspase-3 or cleaved PARP-1 antibody [18,21,36,37]. Indeed, our results suggested a significant increase in the expression levels of cleaved caspase-3 and cleaved PARP-1 in hepatocytes that were neighbouring cancer cells in vessel co-opting RHGP lesions (Figure 1a,b). Conversely, we observed a lower expression of both proteins in adjacent hepatocytes of angiogenic DHGP lesions. To further validate our results and confirm that the hepatocytes were the main source of both cleaved caspase-3 and cleaved PARP-1 in vessel co-opting lesions, we performed co-immunofluorescent (co-IF) staining for chemonaive CRCLM samples (*n* = 3 DHGP; *n* = 3 RHGP) with hepatocyte-specific antigen (HSA) and cleaved caspase-3 or cleaved PARP-1 antibodies. As shown in Figure 1c,d, both cleaved caspase-3 and cleaved PARP-1 were primarily localized in the hepatocytes. Altogether, our data proposed the upregulation of proapoptotic biomarkers in the hepatocytes of vessel co-opting lesions, specifically, the hepatocytes in close proximity to cancer cells.

### 3.2. Cancer Cells Stimulate Apoptosis in Hepatocytes In Vitro

**Figure 2:**
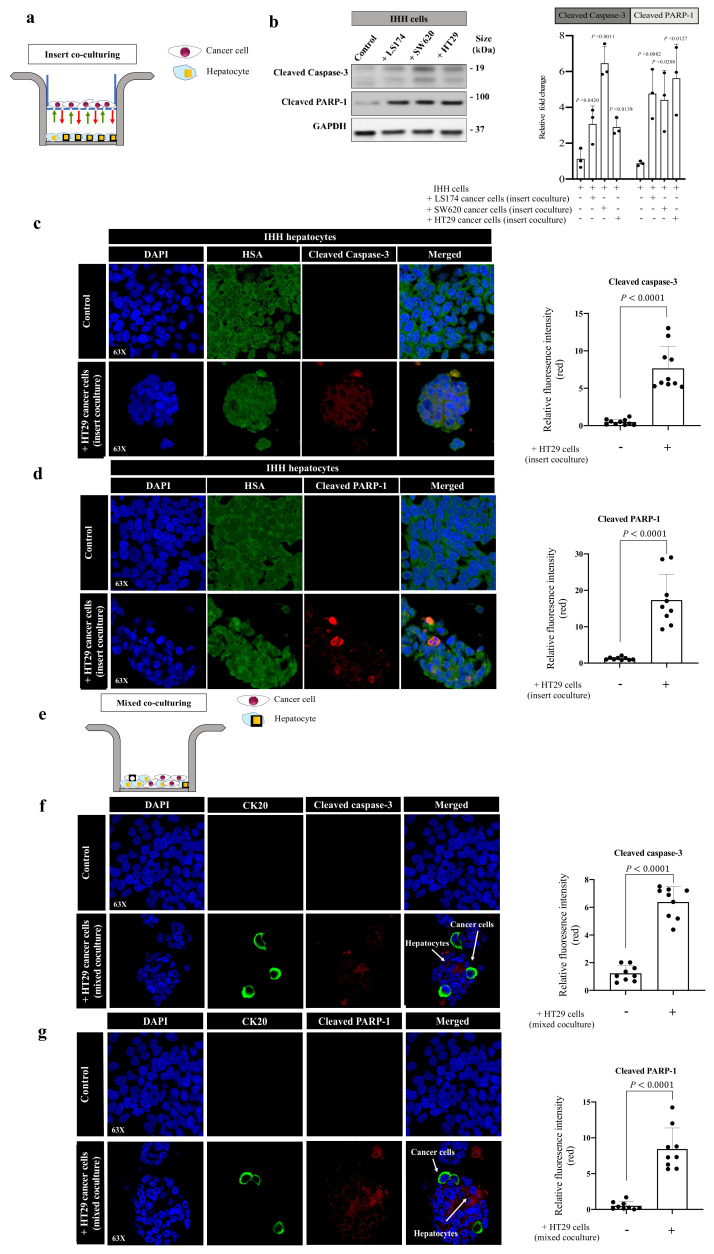
Cancer cells promote apoptosis in hepatocytes in vitro. (**a**) Schematic illustration of indirect (insert) coculturing system. (**b**) The left panel shows immunoblotting of cleaved caspase-3 and cleaved PARP-1 in IHH hepatocytes in the presence or absence of indirect contact with colorectal cancer (LS174, SW620 or HT29) cells. The right panel represents the intensity of cleaved caspase-3 and cleaved PARP-1 bands in three independent experiments. (**c**,**d**) represent co-immunostaining of IHH hepatocytes indirectly cocultured with HT29 cancer cells using hepatocytes marker, HSA (hepatocyte-specific antigen, green), combined with either cleaved caspase-3 or cleaved PARP-1 (left panel). The right panels represent the quantification of positive pixels. Average pixel intensity was measured from three randomly selected areas for each sample. Results are representative of 3 independent experiments. (**e**) Schematic showing mixed (direct) coculturing system. (**f**,**g**) Representative images of coimmunostaining for IHH hepatocytes cocultured with HT29 cancer cells using a mixing coculturing system. The cells were stained with cancer cell marker, CK20 (cytokeratin 20, green), combined with either cleaved caspase-3 or cleaved PARP-1 (red). The right panels represent the quantification of positive pixels. Average pixel intensity was measured from three randomly selected areas for each sample. Results are representative of 3 independent experiments. The bar chart shows mean values ± SD.

To examine whether cancer cells are responsible for the high expression of pro-apoptotic markers in hepatocytes, we assessed the expression of proapoptotic markers (cleaved caspase-3 and cleaved PARP-1) in immortalized human hepatocytes (IHH) upon their indirect contact with colorectal cancer (LS174, SW620 or HT29) cells using the insert coculturing approach (Figure 2a). IHH cells are immortalized human hepatocytes that retain various differentiation phenotypes of primary hepatocytes [38,39,40]. Remarkably, we demonstrated a significant increase in the expression levels of both proapoptotic markers in cocultured IHH hepatocytes with cancer cells (Figure 2b and Figure S5). We also found similar results when we performed immunofluorescence using HSA and cleaved caspase-3 (Figure 2c) or cleaved PARP-1 (Figure 2d) antibodies. Next, we used a direct coculturing system between IHH hepatocytes and HT29 cancer cells to further validate our results (Figure 2e). In this experiment, we used cytokeratin 20 (CK20) [11,32] as a biomarker of colorectal cancer cells, as well as antibodies against cleaved caspase-3 or cleaved PARP-1. The expression levels of both cleaved caspase-3 (Figure 2f) and cleaved PARP-1 (Figure 2g) were significantly increased in the cocultured hepatocytes compared to control, specifically, in the hepatocytes that were in direct contact with cancer cells. Importantly, we also found a significant reduction in the IHH hepatocyte viability upon coculturing with colorectal cancer (LS174, SW620 or HT29) cells (Supplementary Figure S1). In sum, these data imply that the interactions between cancer cells and hepatocytes upregulate the expression of proapoptotic markers in hepatocytes.

It has been reported that tumour cells stimulate autophagy in neighbouring cells of the tumour microenvironment to support their progression [41,42]. Thus, we questioned whether cancer cells in vessel co-opting CRCLM lesions stimulate autophagy in the adjacent hepatocytes to occupy their space. To address this question, we assessed functional autophagy in chemonaive CRCLM specimens (*n* = 5 DHGP; *n* = 5 RHGP) by immunohistochemistry using LC3B and p62/sequestosome 1 (SQSTM1). LC3B and p62 are commonly used as autophagy biomarkers for evaluating autophagy activity [43,44,45]. LC3B is associated with autophagosome formation, while p62 serves as a selective autophagy substrate, which interacts with the autophagy machinery as an adaptor for the target cargo [45,46]. Therefore, the upregulation of LC3B and degradation of p62 can be used as a marker of autophagy [43,44,45]. We demonstrated no significant differences in the expression of LC3B between hepatocytes of vessel co-opting and angiogenic lesions (Supplementary Figure S2a). However, we noticed a moderate positive staining of LC3B in some hepatocytes that were predominantly located at the adjacent parenchyma of vessel co-opting lesions. Our results also exhibited no significant difference in p62 staining between the liver parenchyma of vessel co-opting and angiogenic lesions (Supplementary Figure S2b). These data indicated that the role of autophagy in hepatocytes displacement was not prominent. 

### 3.3. Cancer Cells Induce EMT and Motility in Hepatocytes

**Figure 3:**
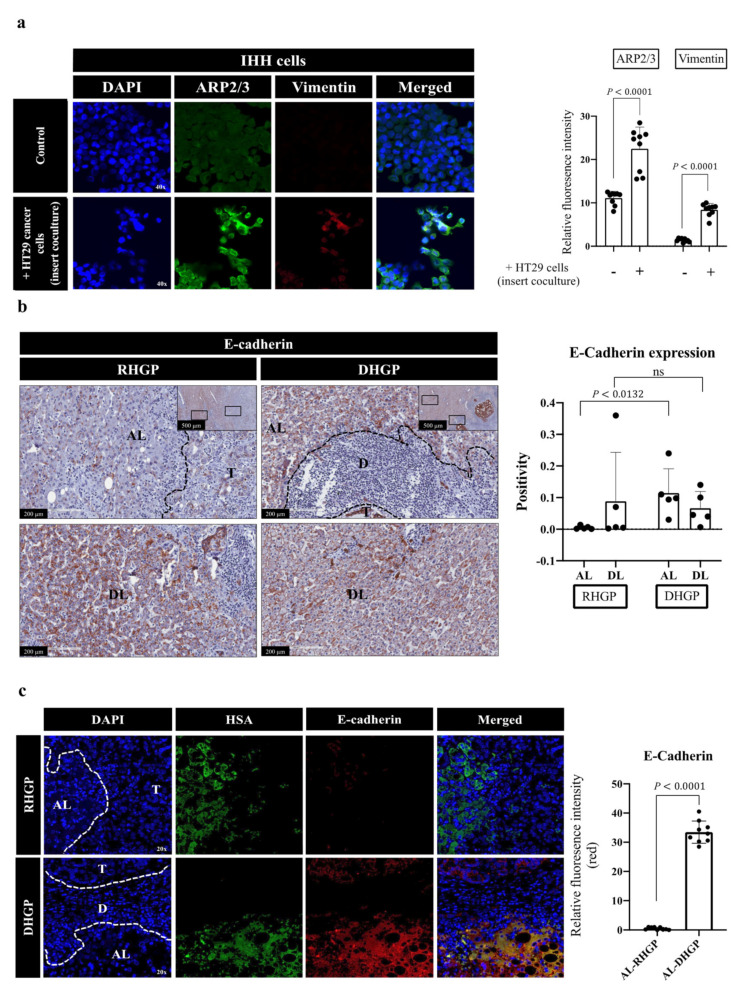
Expression of EMT biomarkers in hepatocytes in vitro and in vivo. (**a**) Representative images of coimmunostaining and colocalization overlay for IHH hepatocytes cocultured (insert coculturing) with HT29 cancer cell lines using motility marker, ARP2/3 (green), and EMT marker, vimentin (red). The right panel shows the quantification of positive pixels. Average pixel intensity was measured from three randomly selected areas for each sample. Results are representative of 3 independent experiments. (**b**) represents immunohistochemical staining for E-Cadherin on FFPE tissue sections of chemonaive CRCLM. The right panels represent quantification of staining positivity that was assessed in RHGP (*n* = 5) and DHGP (*n* = 5) lesions using Aperio software. (**c**) Representative images of coimmunostaining for showing HSA (green) and E-Cadherin (red) on FFPE tissue sections of CRCLM resected from chemonaive patients. The right panels represent the quantification of positive pixels in CRCLM specimens, including RHGP (*n* = 3) and DHGP (*n* = 3). Average pixel intensity was measured from three randomly selected areas for each sample. AL—adjacent liver; D—desmoplastic ring; DL—distal liver; T—tumour. The bar chart shows mean values ± SD. ns = Not significant.

**Figure 4:**
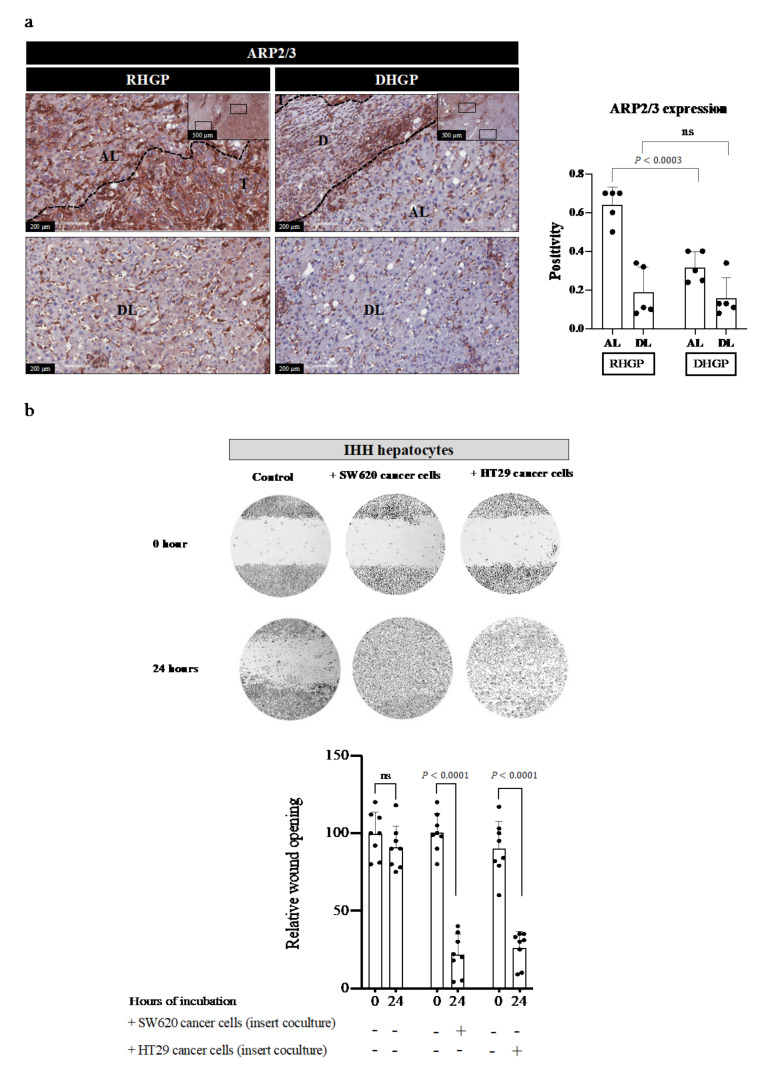
Hepatocyte motility increased upon their interaction with cancer cells. (**a**) shows immunohistochemical staining for motility biomarker (ARP2/3) antibody on FFPE tissue sections of chemonaive CRCLM. The right panel represents quantification of staining positivity that was assessed in RHGP (*n* = 5) and DHGP (*n* = 5) lesions using Aperio software. AL—adjacent liver; D—desmoplastic ring; DL—distal liver; T—tumour. (**b**) Showing scratch assay in IHH hepatocytes upon coculturing (insert coculturing) with colorectal cancer (HT29 or SW620) cells. The experiment was performed in the biological replicate (three wells per experiment). The wound opening was measured and analyzed at 0 and 24 h using image J software (bottom panel). The bar chart shows mean values ± SD. ns = Not significant.

Our coculturing data in Figure 2 suggested alterations in the morphology and arrangement of the hepatocytes upon their interaction with cancer cells. Thus, we hypothesized that hepatocytes that are in close contact with cancer cells may acquire a mesenchymal phenotype, which is known to stimulate EMT-derived migration [30,32]. To test our hypothesis, we cocultured IHH hepatocytes with HT29 colorectal cancer cells followed by immunofluorescence co-staining for EMT and motility biomarkers, using vimentin [47] and actin-related protein 2/3 (ARP2/3) [32,48], respectively. Intriguingly, we observed a significant increase in the expression of both markers in hepatocytes that cocultured (insert coculturing) with cancer cells (Figure 3a). Next, we evaluated EMT in CRCLM lesions (*n* = 5 DHGP; *n* = 5 RHGP) by immunohistochemical (IHC) staining using the E-cadherin antibody. E-cadherin is a key element of maintaining the epithelial phenotype of cells and its loss is correlated with induced EMT [49]. Our IHC staining demonstrated a significant reduction in E-cadherin protein at the adjacent liver parenchyma of vessel co-opting RHGP lesions in comparison to DHGP lesions (Figure 3b). To corroborate our findings, we performed immunofluorescent co-staining for chemonaive CRCLM specimens (*n* = 3 DHGP; *n* = 3 RHGP) using HSA and E-cadherin antibodies. Our data showed that the majority of adjacent hepatocytes to the tumour nests in angiogenic lesions expressed E-cadherin (Figure 3b). In contrast, we observed significantly lower levels of E-cadherin in the adjacent hepatocytes in vessel co-opting lesions. It is worth mentioning that the cancer cells in angiogenic lesions also expressed higher levels of E-cadherin compared to their vessel co-opting counterparts (Figure 3b). These data indicated that the adjacent hepatocytes in vessel co-opting lesions acquired a mesenchymal phenotype. Importantly, the expression levels of ARP2/3 in the hepatocytes of vessel co-opting RHGP lesions were significantly increased (Figure 4a). Nevertheless, our scratch assay experiment [11,32] confirmed that coculturing (insert coculturing) cancer (SW620 or HT29) cells with IHH hepatocytes significantly induced the motility of hepatocytes (Figure 4b). Taken together, our data proposed that cancer cell interactions with hepatocytes augment EMT and motility in hepatocytes. 

### 3.4. RUNX1 Is a Key Player in the Interactions between Cancer Cells and Hepatocytes

**Figure 5:**
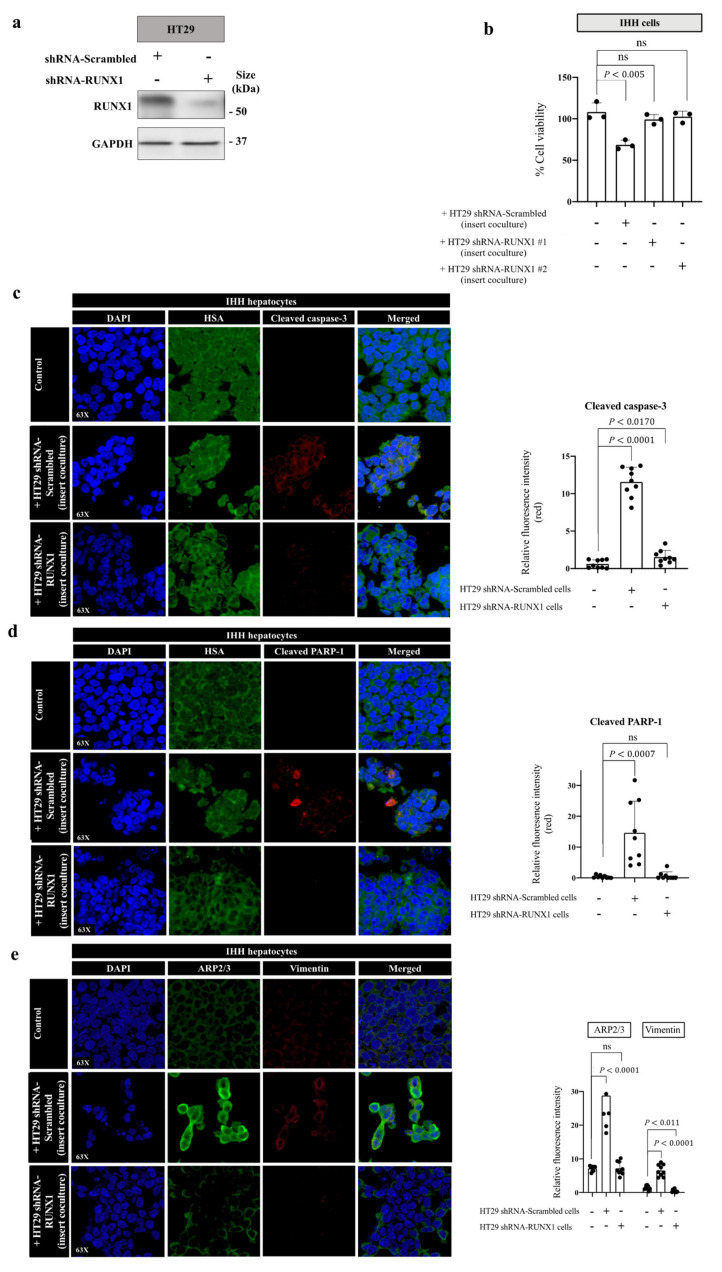
The expression of RUNX1 in cancer cells is crucial for interactions between cancer cells and hepatocytes. (**a**) represents immunoblotting of RUNX1 in HT29 cancer cells expressing either shRNA-scrambled or shRNA-RUNX1. (**b**) Cell viability was assessed with MTT assay in IHH cells cocultured with HT29 colorectal cancer cells expressing shRNA-scrambled or shRNA-RUNX1. (**c**–**e**) represent co-IF of IHH hepatocytes cocultured with HT29 cancer cells expressing shRNA control or shRNA against RUNX1. The cells were stained with HSA and cleaved caspase-3, HSA and cleaved PARP-1, or ARP2/3 and vimentin, respectively. Average pixel intensity was measured from three randomly selected areas for each sample. The right panels represent the quantification of positive pixels. Results are representative of 3 independent experiments. The bar chart shows mean values ± SD. ns = Not significant.

Related transcription factor-1 (RUNX1) is a transcriptional factor that controls the transcription of thousands of genes involved in tumour progression [50], as well as angiogenic [51,52,53] and nonangiogenic tumour vascularization [54,55], including vessel co-option [32]. Recently, we found RUNX1 as a key mediator of vessel co-option development in the CRCLM [32]. Accordingly, the cancer cells of vessel co-opting lesions were characterized by high expression levels of RUNX1, which was associated with an increased motility and EMT [32]. In light of these findings, we hypothesized that the presence of RUNX1 in cancer cells may also play a role in cancer cell-driven hepatocyte phenotype alterations. To test our hypothesis, we generated HT29 cancer cells expressing either control shRNA or shRNA against RUNX1 (Figure 5a and Figure S6). We then performed insert coculturing between the generated HT29 cells and IHH hepatocytes. After 48 h, cell viability was determined in cocultured hepatocytes using an MTT assay. Interestingly, silencing RUNX1 in the cocultured cancer cells abrogated the cytotoxicity of cancer cells against hepatocytes (Figure 5b). Moreover, our IF staining showed that the expression of both cleaved caspase-3 and cleaved PARP-1 in hepatocytes appeared to be dependent on the presence of RUNX1 in the cocultured cancer cells (Figure 5c,d). Similar results were obtained when we cocultured hepatocytes directly with RUNX1-silenced HT29 cancer cells (Supplementary Figure S3a,b). Our IF results also suggested that the presence of RUNX1 in the cancer cells was essential to induce motility and EMT in the adjacent hepatocytes in vitro. As shown in Figure 5e, coculturing RUNX1-silenced HT29 cancer cells with IHH hepatocytes abolished the expression of ARP2/3 and vimentin in the hepatocytes. 

**Figure 6:**
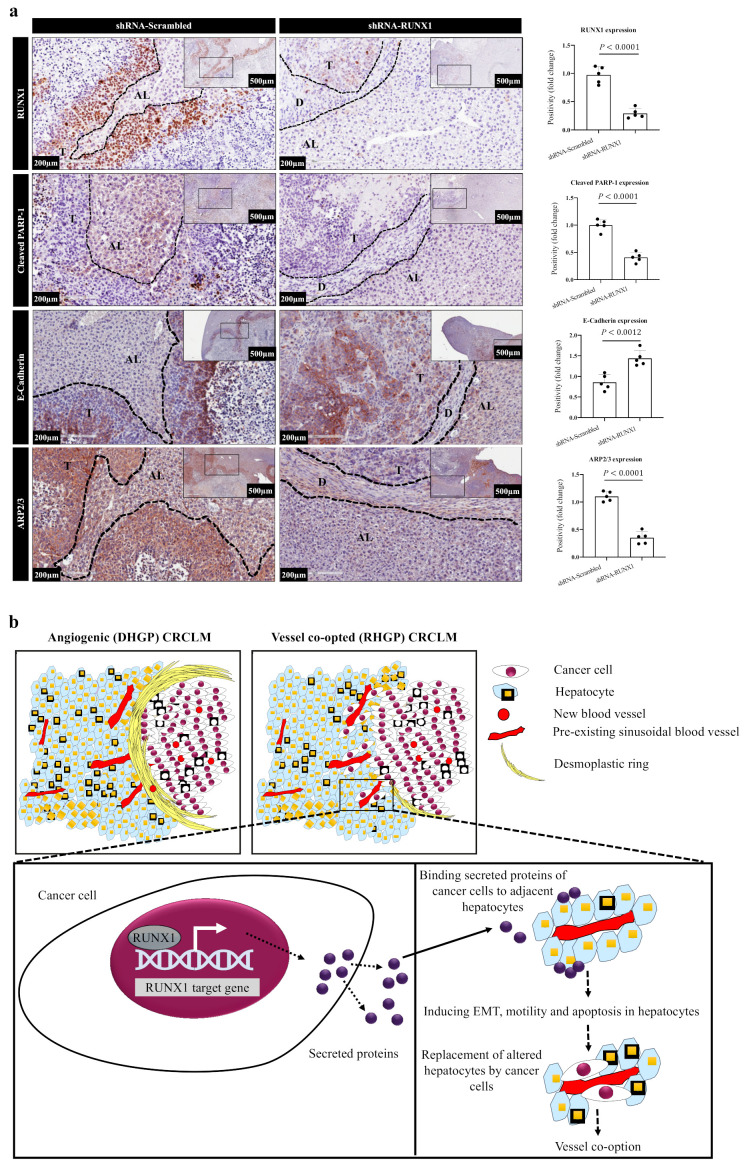
RUNX1 plays a critical role in the interaction between cancer cells and hepatocytes in vivo. (**a**) represents immunohistochemical staining of liver metastatic tumour sections that were generated using HT29 cancer cells expressing shRNA control or shRNA against RUNX1. AL—adjacent liver; D—desmoplastic ring; DL—distal liver; T—tumour. The right panels represent quantification of staining positivity that was assessed in the specimens using Aperio software. (**b**) Schematic illustration showing the main findings in the current study. RUNX1 plays a critical role in the process of hepatocyte displacement by cancer cells. RUNX1 upregulation results in overexpression of its target genes. The expression and secretion of these proteins by cancer cells may stimulate apoptosis, EMT and/or motility of hepatocytes in the surrounding liver. Through these processes, cancer cells induce hepatocyte displacement and replacement around the pre-existing blood vessels and occupy their space to form vessel co-option.

To further ascertain the effect of RUNX1 on hepatocytes alterations, we used the tumour specimens that we previously generated from the intrahepatic xenograft mouse model [32]. These hepatic lesions were generated by injecting HT29 cancer cells expressing scrambled or RUNX1 shRNA into SCID beige mice intrahepatically as explained in Rada et al.’s publication [32]. Of note, our previous publication [32] had reported that the RUNX1-silenced cancer cells were less capable of generating liver metastasis, as well as forming vessel co-opting lesions compared to control cancer cells. To examine whether the absence of RUNX1 in the cancer cells affects cancer cell-driven hepatocyte modifications in vivo, we stained specimens that were generated from our previous publication [32] with PARP-1, ARP2/3, or E-cadherin antibody. Interestingly, we observed a lower expression of cleaved PARP-1 and ARP2/3 in the hepatocytes of metastatic lesions that were generated from RUNX1-deficient HT29 cancer cells in comparison to those that were generated by control HT29 cancer cells (Figure 6a). In contrast, E-cadherin was highly expressed in the hepatocytes of RUNX1-silenced metastatic lesions (Figure 6a).

It is worth mentioning that we previously reported the upregulation of TGFβ1 in vessel co-opting CRCLM lesions [32]. Accordingly, RUNX1 played a key role in the expression of TGFβ1 through its target gene thrombospondin 1 (TSP1) in vessel co-opting CRCLM [32]. Importantly, TGFβ1 was reported as an inducer of apoptosis, EMT, and motility in various cells, including hepatocytes [56]. As shown in Supplementary Figure S4a and Figure S7, the conditioned media of cocultured IHH hepatocytes with cancer (LS174, SW620 or HT29) cells showed a higher abundance of TGFβ1 compared to the control. Remarkably, our immunoblotting data suggested an overexpression of TGFβ1 in cocultured IHH hepatocytes (Supplementary Figure S4b) and cancer cells (Supplementary Figure S4c). However, further studies are required to determine the main source of TGFβ1. 

To identify the effect of TGFβ1 on phenotypic alterations in hepatocytes, we exposed IHH hepatocytes to 100 picomolar recombinant TGFβ1 for 24 h. We observed the overexpression of cleaved caspase-3, cleaved PARP-1, vimentin, and ARP2/3 in IHH hepatocytes upon exposure to recombinant TGFβ1 (Supplementary Figure S4d). Overall, our results confirmed the governing role of RUNX1 in cancer cell-driven phenotype modifications in hepatocytes, which facilitated hepatocyte displacement (Figure 6b). However, further investigations are needed to identify other relevant molecules that mediate cancer cell-dependent phenotypic alterations in hepatocytes. 

## 4. Discussion

We previously hypothesized that vessel co-option occurs in four different fluid phases in CRCLM: (a.) increase in the motility of cancer cells to infiltrate surrounding parenchyma; (b.) displacement of the hepatocytes at the edge of tumour nests and occupation of their space by cancer cells; (c.) physical contact between cancer cells and sinusoidal vessels; (d.) forming immune microenvironment that favours vessel co-option [13]. Our team reported the molecular signaling of cancer cell motility in vessel co-opting lesions, which are mainly associated with RUNX1 [32] and ARP2/3 [8,32] overexpression. However, the pathways underlying hepatocyte displacement were not investigated yet. Herein, our extensive studies including in vitro and in vivo analyses uncovered the molecular pathways that contribute to hepatocyte displacement, which involves apoptosis, EMT, and motility. However, the question of whether these processes proceed concurrently or consecutively requires additional investigation. 

Various studies have investigated the role of apoptosis in nonangiogenic and antiangiogenic resistant tumours [22,23,57,58]. These studies have mainly focused on the role of apoptosis in cancer cells instead of their microenvironment. In this context, it has been reported that proapoptotic genes, including FOS, FAH, and PRODH, are upregulated in the cancer cells of nonangiogenic NSCLC [22]. On the other hand, high levels of serpin B2 (SERPINB2) have been demonstrated in the cancer cells of vessel co-opting brain metastatic lesions, which play a key role in apoptosis suppression [57,58]. These findings were consistent with our results, which suggested low expression levels of proapoptotic biomarkers (cleaved caspase-3 and cleaved PRAP-1) in the cancer cells of CRCLM lesions. 

EMT is a pivotal process that is implicated in invasiveness and metastatic behaviour of epithelial cancers, and it also correlates with poor clinical outcomes in several solid tumours [59]. EMT has been shown to increase cancer cell motility that favours invasion and dissemination [30,60]. It has been suggested that partial EMT is associated with tumour resistance to chemotherapy [61] and antiangiogenic treatments [62,63]. Of note, vessel co-opting tumours are also known to be resistant to antiangiogenic therapy [8]. We recently demonstrated that both EMT and motility are essential for cancer cell infiltration within liver parenchyma in CRCLM [32]. In the current study, we also found a positive correlation between EMT and motility in hepatocytes and vessel co-option. 

The cancer cells of vessel co-opting CRCLM lesions were characterized by a high expression of RUNX1 that correlated with the upregulation of its target genes, including TSP1 [32]. Accordingly, the secreted TSP1 by cancer cells augmented the expression and activation of TGFβ1 in the hepatocytes in their neighbouring hepatocytes [32]. In this study, our data proposed RUNX1 and TGFβ1 as key mediators of hepatocyte displacement. In agreement with our data, TGFβ1 has been shown to induce apoptosis [64] and EMT [65,66]. Despite the lack of literature that suggests a positive correlation between TGFβ1 and hepatocyte motility, our in vitro data confirmed that exposing hepatocytes to TGFβ1 leads to the upregulation of the motility marker (ARP2/3) in hepatocytes.

Our study suggests that cancer cells stimulate apoptosis and EMT in hepatocytes through the overexpression of TGFβ1. Then, a question arises as to whether these two processes occurred simultaneously or sequentially? In agreement with our study, it has been reported that TGFβ1 can stimulate apoptotic and EMT responses in AML-12 mouse hepatocytes in a dose- and time-dependent manner [67]. Accordingly, the net effect of TGFβ1 was contingent on the cellular context and the specific state of cells, such as the genetic basis and cell cycle state. Therefore, further investigation needs to determine the reasons underlying the differential response of hepatocytes to cancer cells in vessel co-opting CRCLM lesions.

In conclusion, our data uncovered RUNX1 and TGFβ1 signaling pathways as major pathways that orchestrate hepatocyte displacement in CRCLM to form vessel co-option, which was arbitrated by apoptosis, EMT, and motility. The presented mechanisms provide further insights into the processes involved in vessel co-option in CRCLM. However, more investigations are needed to address all mediators that contribute to hepatocyte displacement, which would be pivotal in finding new strategies to overcome the development of vessel co-option and resistance to antiangiogenic therapy in CRCLM. 

## Data Availability

The data presented in this study are available within the article.

## Supplementary Materials

The following supporting information can be downloaded at: https://www.mdpi.com/article/10.3390/cancers14051318/s1, Figure S1: Effect of cancer cells on viability in hepatocytes, Figure S2: Expression of autophagy markers in chemonaive CRCLM specimens, Figure S3: The role of RUNX1 in cancer cell-dependent hepatocyte apoptosis, Figure S4: TGFβ1 mediates cancer cell-driven hepatocytic alterations in vitro, Figure S5: Original uncropped western blots of Figure 2b, Figure S6: Original uncropped western blots of Figure 5a, Figure S7: Original uncropped western blots of Figure S4.
