# Supplementary material for: Cancer Cells Promote Phenotypic Alterations in Hepatocytes at the Edge of Cancer Cell Nests to Facilitate Vessel Co-Option Establishment in Colorectal Cancer Liver Metastases
Source: Cancers (Basel). 2022 Mar 4;14(5):1318. doi: 10.3390/cancers14051318 (PMC8909291; doi:10.3390/cancers14051318)
Supplement: Supplementary file 1 [file cancers-14-01318-s001.zip › cancers-1530610-supplementary.pdf]

Cancer Cells Promote Phenotypic Alterations in Hepatocytes at the Edge of Cancer Cell Nests to Facilitate Vessel Co-Option Establishment in Colorectal Cancer Liver Metastases

Miran Rada, Migmar Tsamchoe, Audrey Kapelanski-Lamoureux, Nour Hassan, Jessica Bloom, Stephanie Petrillo, Diane H. Kim, Anthoula Lazaris and Peter Metrakos

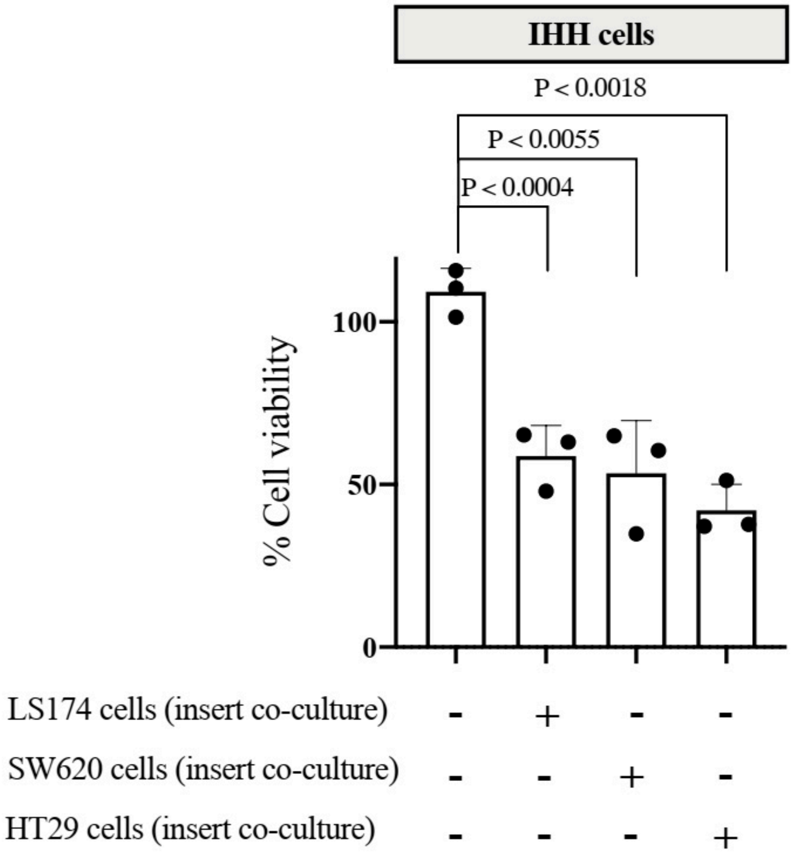

Figure S1. Effect of cancer cells on viability in hepatocytes. The MTT assay was used to examine the effect of various colorectal cancer (LS174, SW620 or HT29) cells on hepatocytes after 48 hours of insert co-culturing. The experiment was performed in three biological replicates. The bar chart shows mean values ± SD.

a

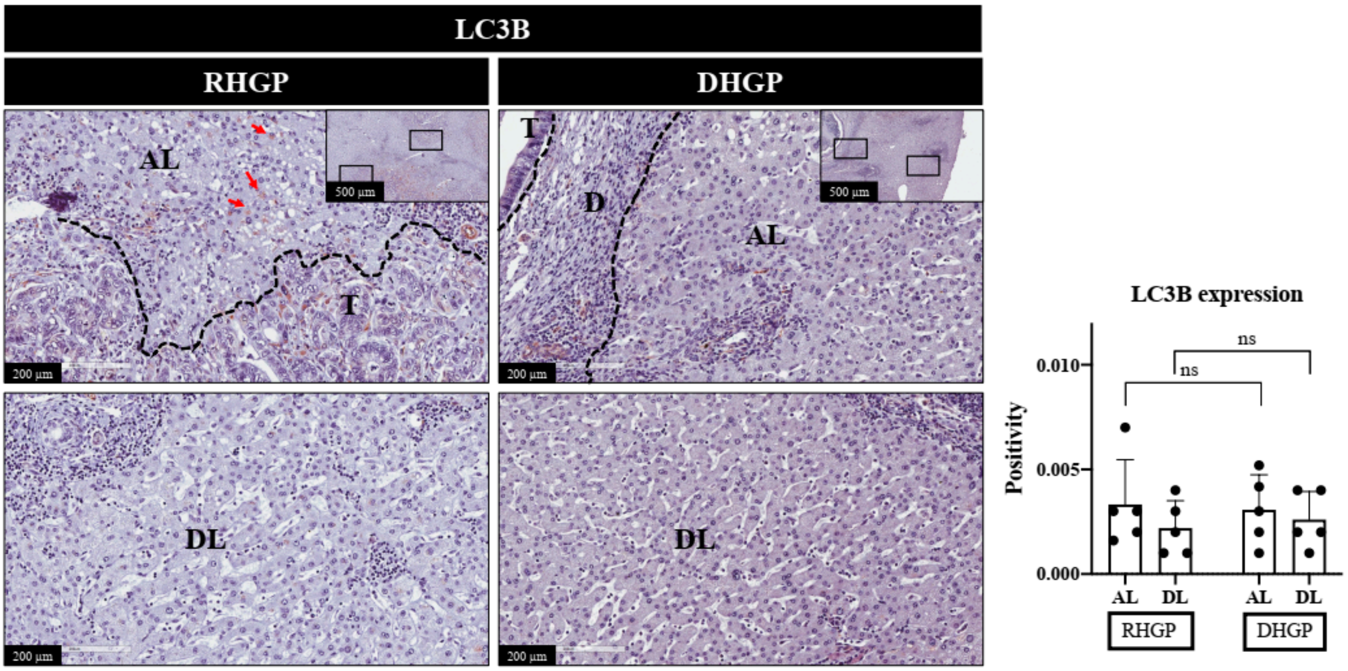

b

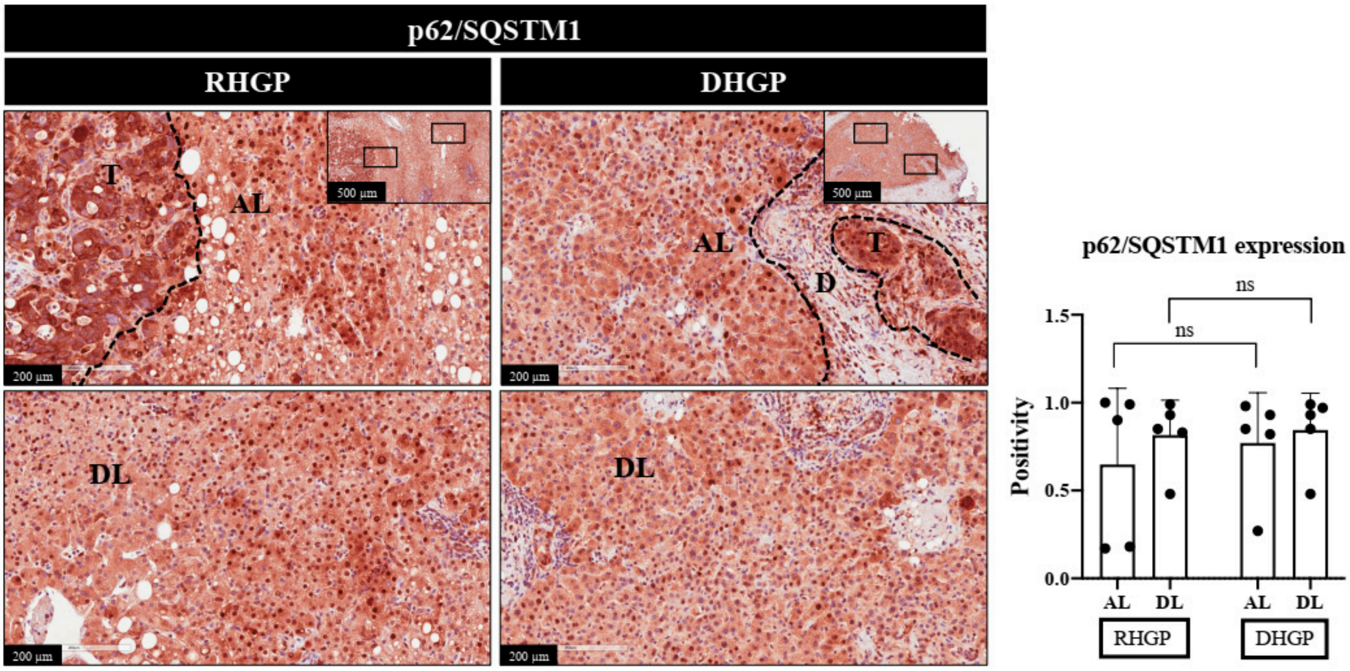

Figure S2. Expression of autophagy markers in chemonaive CRCLM specimens. **a** and **b**. Immunohistochemical staining of chemonaïve CRCLM specimens using anti-LC3B or anti-p62 antibody (left panels). The right panels represent quantification of staining positivity that assessed in RHGP (n = 5) and DHGP (n = 5) lesions using an optimized Aperio algorithm. AL = Adjacent liver, D: Desmoplastic ring, DL = Distal liver, T: Tumour.

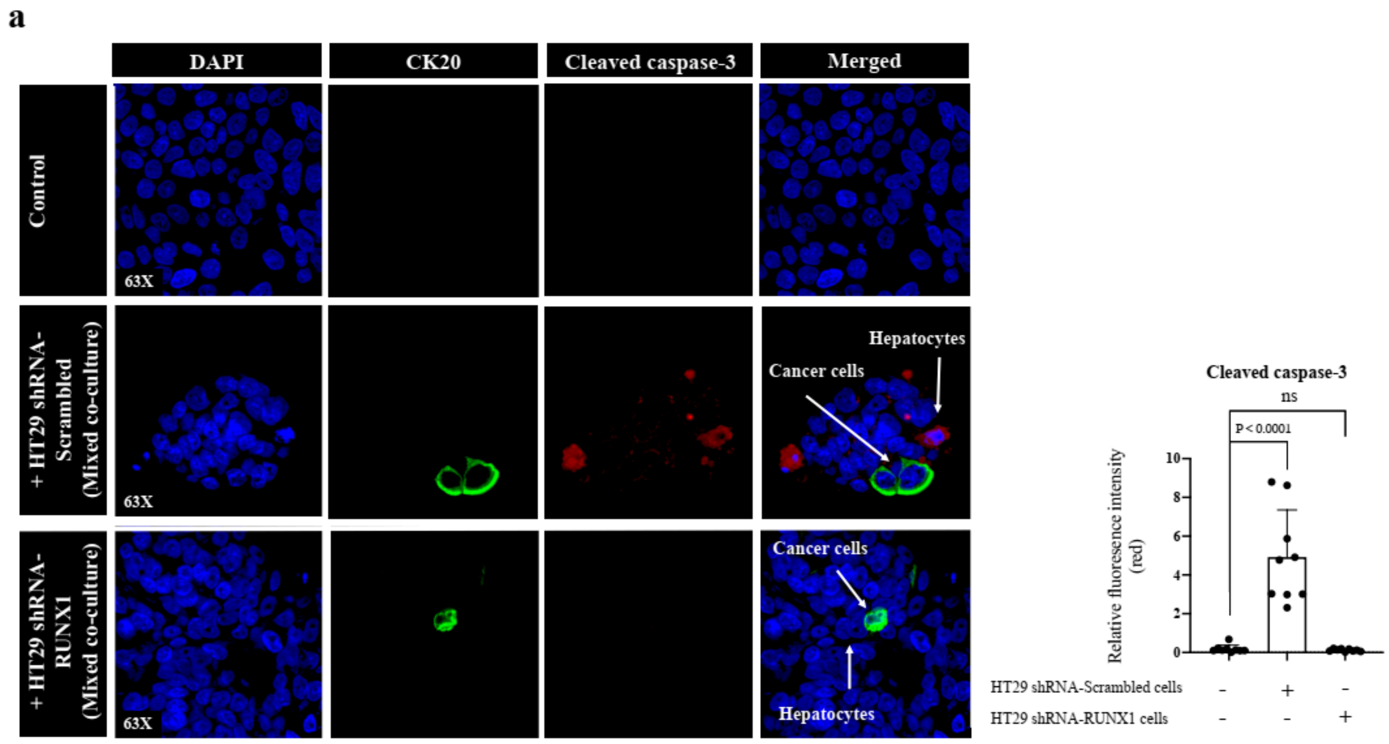

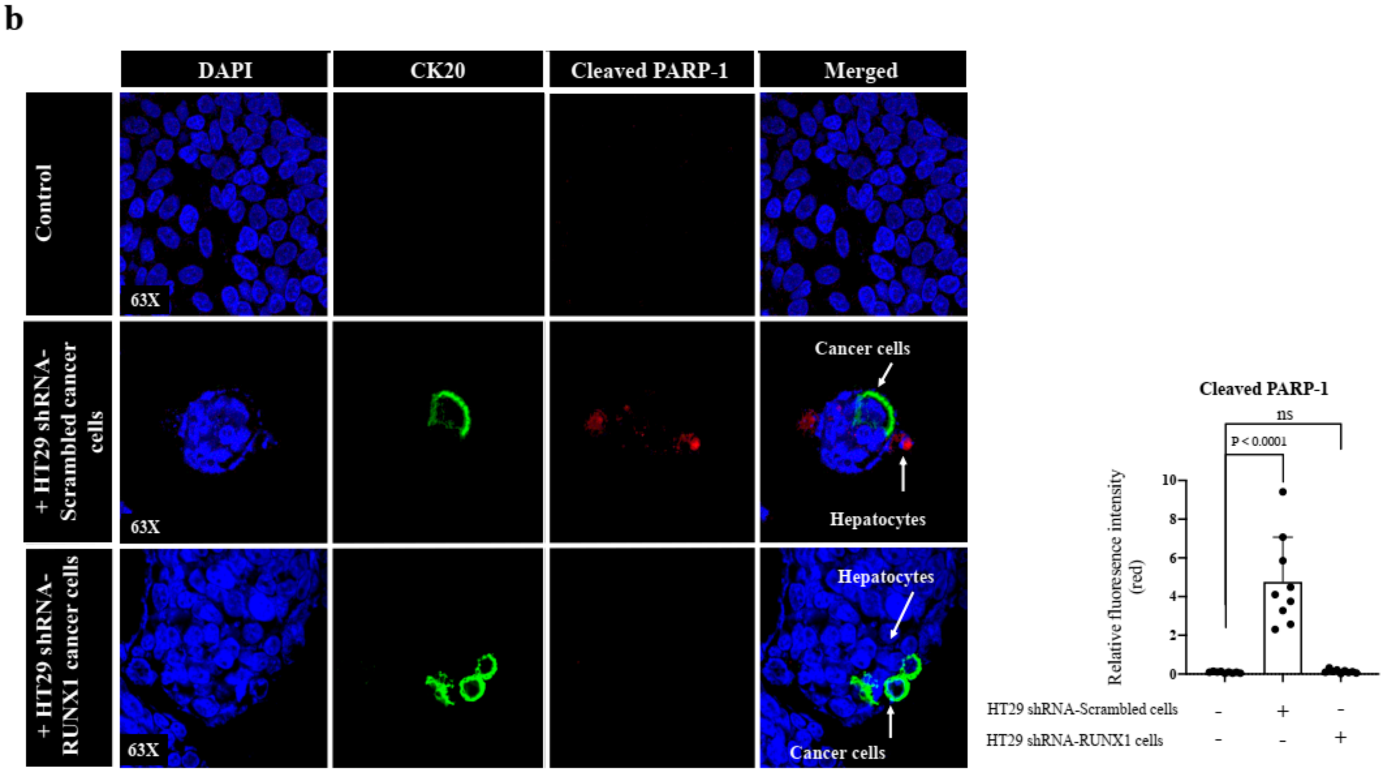

Figure S3. The role of RUNX1 in cancer cell-dependent hepatocyte apoptosis. **a** and **b**. Represent co-IF staining of IHH hepatocytes co-cultured with HT29 cancer cells expressing shRNA control or shRNA against RUNX1. The cells were stained cancer cells biomarker (CK20) and cleaved caspase-3 or cleaved PARP-1 respectively. The right panels represent the quantification of positive pixels. Average pixel intensity was measured from three randomly selected areas for each sample. The experiment was performed in three biological replicates. The bar chart shows mean values ± SD.

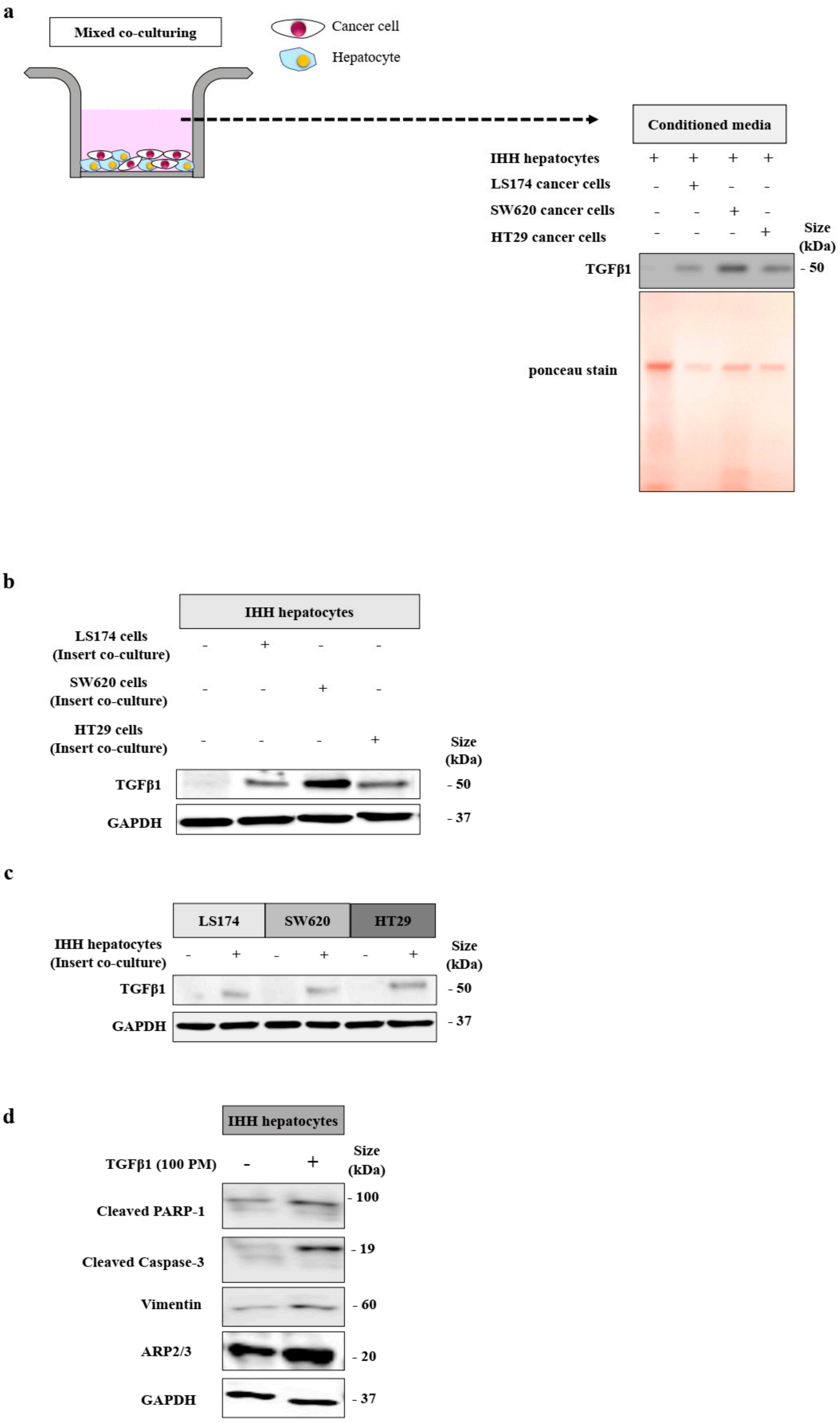
a
Mixed co-culturing
Cancer cell
Hepatocyte
Conditioned media
IHH hepatocytes + + + +
LS174 cancer cells - + - -
SW620 cancer cells - - + -
HT29 cancer cells - - - +
Size (kDa)
TGFβ1
- 50
ponceau stain
b
IHH hepatocytes
LS174 cells (Insert co-culture) - + - -
SW620 cells (Insert co-culture) - - + -
HT29 cells (Insert co-culture) - - - +
Size (kDa)
TGFβ1
- 50
GAPDH
- 37
c
LS174
SW620
HT29
IHH hepatocytes (Insert co-culture) - + - + - +
Size (kDa)
TGFβ1
- 50
GAPDH
- 37
d
IHH hepatocytes
TGFβ1 (100 PM) - +
Size (kDa)
Cleaved PARP-1
- 100
Cleaved Caspase-3
- 19
Vimentin
- 60
ARP2/3
- 20
GAPDH
- 37

Figure S4. TGFβ1 mediates cancer cell-driven hepatocytic alterations in vitro. **a.** Left panel represents schematic of experimental design. The right panel shows the abundance of TGFβ1 in the conditioned media of IHH hepatocytes that co-cultured with various cancer cells. Ponceau staining was used as a loading control. **b.** Immunoblotting for TGFβ1 was performed in control and co-cultured IHH hepatocytes with colorectal cancer (LS174, SW620 or HT29) cells. **c.** Immunoblotting represents TGFβ1 expression in LS174, SW620 or HT29 cancer cells in the presence or absence of co-cultured IHH hepatocytes. **d.** Immunoblotting shows cleaved caspase-3, cleaved PARP-1, vimentin, and ARP2/3 expression in IHH hepatocytes that were exposed to recombinant TGFβ1 for 24 hours. The experiment was performed in three biological replicates.

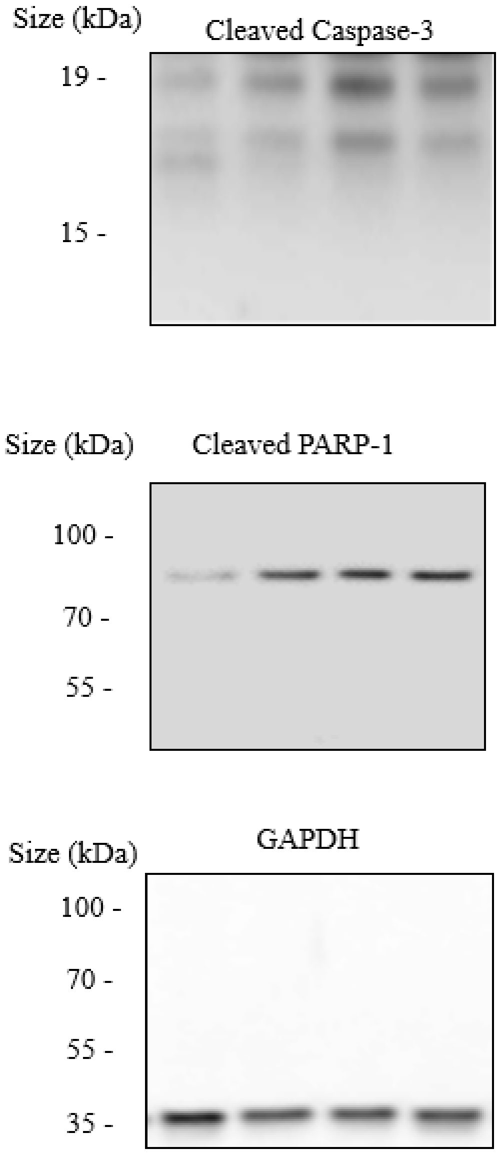

Figure S5. Original uncropped western blots of figure 2b.

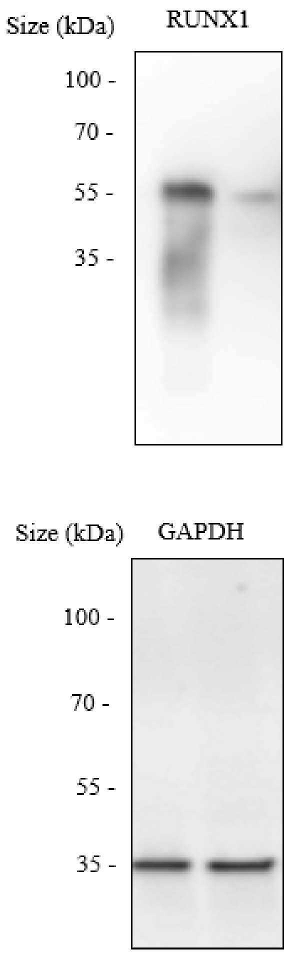

Figure S6. Original uncropped western blots of figure 5a.

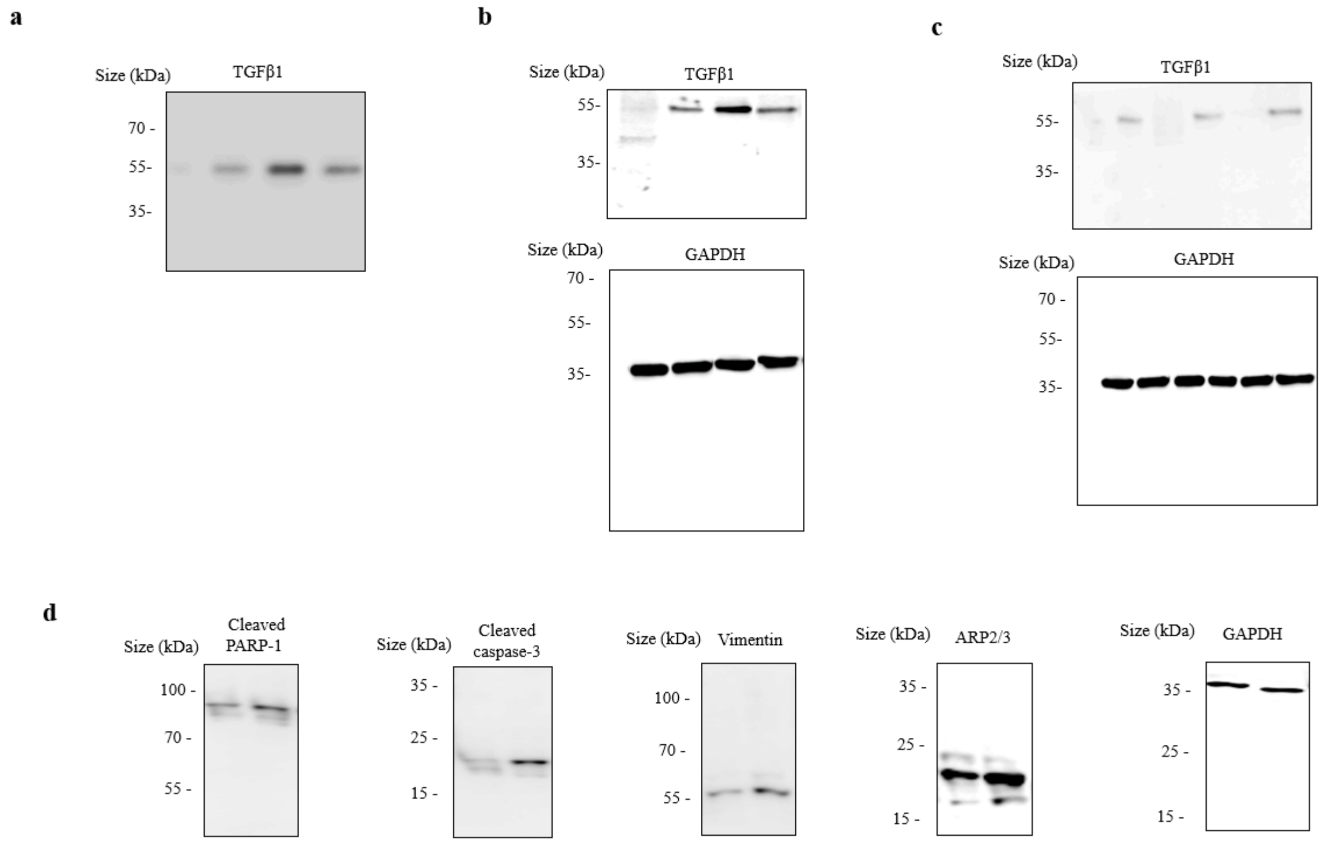

Figure S7. Original uncropped western blots of Figure S4.
